# Rehabilitation Practices Delivered by Physical and Occupational Therapists to Brazilian Children With Congenital Zika Syndrome: A Cross-Sectional Study

**DOI:** 10.9745/GHSP-D-23-00219

**Published:** 2023-12-22

**Authors:** Kennea Martins Almeida Ayupe, Ianka Maria Bezerra Cunha Dias, Ana Paula Martins Cazeiro, Ana Carolina de Campos, Egmar Longo

**Affiliations:** a Department of Integrated Health Education, Center for Health Sciences, Universidade Federal do Espírito Santo, Vitória, Espírito Santo, Brazil.; b Postgraduate Program in Rehabilitation Sciences, Universidade Federal do Rio Grande do Norte, Faculty of Health Sciences of Trairi, Santa Cruz, Rio Grande do Norte, Brazil.; c Faculty of Education, Universidade Federal do Ceará, Fortaleza, Ceará, Brazil.; d Postgraduate Program in Physical Therapy, Universidade Federal do São Carlos, São Carlos, São Paulo, Brazil.

## Abstract

**Introduction:**

Congenital Zika syndrome (CZS) is a health condition that has affected the development of thousands of children in Brazil. Because it is a new condition, its understanding is an ongoing process. Therefore, it is important to know the rehabilitation interventions being delivered to improve the functioning of these children. We aimed to describe the practices of physical therapists (PTs) and occupational therapists (OTs) who provide follow-up care for children with CZS in Brazil.

**Methods:**

This cross-sectional study included PTs and OTs who assist children with CZS in Brazil. An online questionnaire was used to verify the participants' personal characteristics and professional work environment, as well as the rehabilitation programs they implemented in Brazil for children with CZS. Data were analyzed using descriptive statistics.

**Results:**

A total of 116 professionals (79 PT and 37 OT) who work mainly in public health services (81.9%) participated in the study. Of these, 24.1% plan interventions based on reading scientific articles, 66.4% did not report using the biopsychosocial model, 52.6% do not perform any assessments before starting an intervention, 31.9% use neurodevelopmental treatment, and 22.4% use sensorimotor stimulation interventions. The majority of the interventions are delivered 1 to 2 times a week, lasting up to 1 hour.

**Conclusions:**

Professional training and knowledge translation strategies are needed to implement evidence-based practices and improve the quality of rehabilitation programs for Brazilian children with CZS.

## Abstract (precis)

This study highlighted that professional training and knowledge translation strategies are needed to implement evidence-based practices and improve the quality of physical and occupational therapy programs for Brazilian children with congenital Zika syndrome.

## Teaser

**Key Findings:**

- Congenital Zika syndrome (CZS) has drastically affected the child development of thousands of children in Brazil, leading to the need for rehabilitation interventions focused on improving functionality, including activity and social participation.
- In Brazil, the care that physical therapists and occupational therapists provide to children with CZS is mostly through the public health service, with frequency and intensity below that recommended by current scientific evidence.
- Most implemented intervention models are based on nonstandard assessments of functionality and on interventions that do not prioritize the improvement of social participation and have little scientific evidence.

**Key Implications:**

- The public health system must support professional training and knowledge translation strategies for the implementation of evidence-based practices in the rehabilitation processes of Brazilian children with CZS.
- Physiotherapists and occupational therapists must consider the cost-effectiveness relationship when choosing interventions delivered to Brazilian children with CZS, mainly because it is a low-income context with limited resources.

## INTRODUCTION

The Zika virus outbreak has caused thousands of cases of congenital Zika syndrome (CZS) in Brazil since 2015.^1^^,^^2^ The syndrome is characterized by fetal brain malformation and microcephaly associated with other conditions, such as hearing, visual, motor, and intellectual disabilities.^1^^,^^3^ According to epidemiological data from the Brazilian Ministry of Health, the majority of confirmed cases of CZS (around 1,711 live births) were born between 2015 and 2017, mainly in the Northeast region, followed by the Southeast region of the country.^4^^,^^5^ Since then, the number of new cases has gradually declined and is now low.^4^ Recently, it was proposed that because CZS has a clinical presentation compatible with a diagnosis of cerebral palsy (CP), it should be consistently recognized under the umbrella term CP, which is a heterogeneous clinical descriptive condition rather than an etiologic diagnosis.^6^ The majority of children with CZS are classified in the Gross Motor Function Classification System as level IV or V, which correspond to the poorest functional levels.^3^^,^^7^^–^^12^ In addition to the typical characteristics of CP, children with CZS commonly present with other signs, such as arthrogryposis, clubfoot, and abnormalities in the cardiac, genitourinary, and gastrointestinal systems.^5^

Despite the significant decrease in the number of new cases, the provision of rehabilitation for children in Brazil with CZS is still challenging. Although there are evidence-based treatments for CP, we do not yet know whether they are being applied to children with CZS.^13^ Currently, interventions with sufficient evidence for improving motor disabilities in children with CP include task orientation, high-intensity practice, family-centered care, and environmental enrichment.^14^^,^^15^ However, a recent scoping review study identified that, in Brazil, research on the rehabilitation of children with CP still does not use these contemporary concepts.^13^ The few Brazilian studies available to date in the literature for rehabilitation in children with CZS are small and have focused on Goals - Activity - Motor Enrichment (GAME),^16^ intensive suit therapy training,^17^ and neurodevelopmental treatment (NDT) (also known as Bobath).^18^

Given the gap in knowledge of interventions applied to the rehabilitation of children with CZS, there is an urgent need to gather information to implement evidence-based practices that also use the biopsychosocial model recommended by the International Classification of Functioning, Disability and Health (ICF).^2^^,^^19^^,^^20^ Even though evidence-based interventions for children with CP may have a positive effect on children with CZS, the efficacy of interventions and theoretical models currently used in clinical practice to improve functioning in this population remain unknown.

Given the gap in knowledge of interventions applied to the rehabilitation of children with CZS, there is an urgent need to gather information for the implementation of evidence-based practices.

Because CZS has caused disabilities in thousands of children and has motivated the creation of public health policies in Brazil,^4^^,^^21^ it is important to understand the delivery of rehabilitation services to these children to inform current and future policies. Therefore, we aimed to verify the practices of physical therapists (PTs) and occupational therapists (OTs) who provide follow-up care for children with CZS in Brazil based on the scientific evidence available for CP and on the ICF model.

## METHODS

### Design and Participants

This is a cross-sectional exploratory study in which PTs and OTs responded to a self-administered online questionnaire. Professionals with active licenses in Brazil and who provide follow-up care for children with CZS were included. To be included, professionals must have been providing follow-up care for children with CZS at the time of study entry, regardless of the number of children or working hours. Professionals who were not currently working with children with CZS or who agreed to participate but did not fill out the questionnaire were excluded. The study was conducted between September 2019 and July 2020.

Participants were recruited through advertisements on social media, on the websites of nongovernmental organizations, and via emails sent to PT and OT national associations and public health departments. The sample was nonprobabilistic; the responses of all professionals who met the inclusion criteria and agreed to participate were included in the data analysis. Considering data from a professional practice-oriented organization (Brazilian Association of Neurofunctional Physiotherapy),^22^ at the time the study was carried out, there were 409 PTs who worked specifically with people with neurological problems throughout Brazil, including in regions without cases of CZS. Brazil lacks associations of OTs working in the neurorehabilitation area; however, based only on the available governmental statistics, they represent approximately 10% of the number of PTs in Brazil.

### Measures and Procedures

An online questionnaire, adapted from Shikako-Thomas et al.,^23^ was designed for data collection (Supplement). Initially, the questionnaire was adapted to assess features, knowledge, attitudes, and practices of PTs and OTs who provide follow-up care for children with CZS in Brazil. Two experienced PTs involved in evidence-based practices with experience in Brazil, the United States, and Europe tested and administered the questionnaire to 5 professionals before being used in the study. Both experts considered the instrument acceptable and easy to use and provided comments that helped to refine it, including questions relevant to the PT and OT work context in Brazil.

The final version of the questionnaire designed for this study consisted of 25 questions divided into 3 sections: (1) 6 questions on personal and professional characteristics (e.g., age and educational level); (2) 7 questions on work environment (e.g., funding sources); and (3) 12 questions on rehabilitation intervention characteristics (e.g., ICF and evidence-based practice knowledge, assessment, intervention types). The questionnaire was shared with potential participants by email as a Google Forms link. Participants used the link to complete the questionnaire and submit it to the administrator. An average time of 30 minutes was sufficient to complete the questionnaire. Participants were included after providing consent; the privacy and confidentiality of personal information were maintained.

### Data Analysis

Data were analyzed using SPSS version 20. A descriptive analysis of the main study variables was performed using absolute and relative frequencies, percentages, and means.

### Ethical Approval

Ethics approval for the study was obtained by the Trairi Faculty of Health Sciences, Federal University of Rio Grande do Norte, Brazil (number 18049919.4.0000.5568).

## RESULTS

Initially, 218 professionals received the email with the link to access the questionnaire and agreed to participate. Of these, 97 (44.5%) did not answer the questionnaire, and 121 (55.5%) participants answered the questionnaire and submitted it to the researchers. Five participants were excluded because they did not work directly in the rehabilitation of children with CZS. Thus, 116 professionals responded to the questionnaire: 79 PTs (68.1%) and 37 OTs (31.9%). Table 1 presents the characteristics of the participants. The majority were female (85.3%), had at least 1 specialization degree (in addition to graduation), and more than 4 years of clinical experience with children. Participants were from the Northeast (68%), Southeast (28%), South (2%), and North (2%) regions. Considering all PTs who work with people with neurological impairments, according to the Brazilian Association of Neurofunctional Physiotherapy data, we observed a response rate of 19.3%.

**TABLE 1.** Characteristics of the Participants in a Study to Explore Follow-Up Care for Children With Congenital Zika Syndrome in Brazil

	Total, No. (%)	Physical Therapist, No. (%)	Occupational Therapist, No. (%)
Participants	116 (100)	79 (68.1)	37 (31.9)
Gender			
Female	99 (85.3)	68 (86.1)	31 (83.8)
Male	17 (14.7)	11 (13.9)	6 (16.2)
Age, years			
20–29	34 (29.3)	22 (27.8)	12 (32.4)
30–39	41 (35.3)	29 (36.7)	12 (32.4)
40–49	33 (28.5)	24 (30.4)	9 (24.4)
>50	8 (6.9)	4 (5.1)	4 (10.8)
Educational level			
Bachelor's degree	25 (21.6)	15 (19.0)	10 (27.0)
Specialist	57 (49.1)	35 (44.3)	22 (59.5)
Master's degree	26 (22.4)	22 (27.8)	4 (10.8)
Ph.D. degree	8 (6.9)	7 (8.9)	1 (2.7)
Clinical experience with children, years			
1–3	28 (24.1)	19 (24.0)	9 (24.3)
4–10	43 (37.1)	27 (34.2)	16 (43.2)
>10	45 (38.8)	33 (41.8)	12 (32.5)

Regarding the work environment for the whole sample, services were mainly supported by public resources (81.9%), and more than half (53.4%) did not conduct research in the field of rehabilitation. The participants worked in public rehabilitation centers/clinics (63.8%), university centers (11.2%), community organization centers (6.9%), and private clinics (18.1%). Thirty percent of the professionals worked in interdisciplinary teams that included physicians, 48.4% worked together with another professional specialty (e.g., speech therapist or music therapist), and 21.6% worked alone.

Table 2 shows the characteristics of the rehabilitation interventions. The minority of all participants (24.1%) planned therapy based on reading scientific articles, and most (52.6%) did not evaluate or did not know how to answer which type of assessment was performed before starting the interventions, especially the PTs (62.0%). The majority of all participants (66.4%) did not use the ICF model to guide the assessment or to plan the intervention. The assessment tool most frequently cited by PTs was the Gross Motor Function Measure (23.3%) and by OTs was the Pediatric Evaluation of Disability Inventory (11.2%). The most frequently used intervention by PTs was NDT (31.9%) and by OTs sensorimotor stimulation (43.2%). Physical therapists provided follow-up care to an average of 4.4 children per week, and OTs provided follow-up care to 5.5 children per week. The intervention frequency varied from 1 (33.6%) to 2 (44.8%) times a week, with an intensity equal to or less than 1 hour per session (54.3%).

**TABLE 2.** Participant Responses to Questionnaire on Rehabilitation Interventions Provided to Children With Congenital Zika Syndrome in Brazil

Questions	Total, No. (%)	Physical Therapist, No. (%)	Occupational Therapist, No. (%)
Which are the sources of information for planning interventions?			
Does not use any sources	64 (55.1)	45 (57.0)	19 (51.3)
Scientific articles	28 (24.1)	18 (22.8)	10 (2.7)
Training courses	15 (12.9)	12 (15.2)	3 (8.1)
Institutional normative instructions	7 (6.0)	3 (3.8)	4 (10.8)
Google/books	2 (1.7)	1 (1.3)	1 (2.7)
Do you use assessment tools/instruments before/after intervention - which ones?			
Does not perform any assessment	61 (52.6)	49 (62.0)	12 (32.4)
Gross Motor Functional Measure	27 (23.3)	23 (29.1)	4 (10.8)
Physical examination without standardized tools	20 (17.2)	12 (15.2)	8 (21.6)
Pediatric Evaluation Disability Inventory	13 (11.2)	8 (10.1)	5 (13.5)
Bayley Scales of Infant development	8 (6.9)	6 (7.6)	2 (5.4)
Denver Developmental Screening Test	5 (4.3)	2 (2.5)	3 (8.1)
Canadian Occupational Performance Measure	5 (4.3)	0 (0.0)	5 (13.5)
Anamnesis only	5 (4.3)	1 (1.3)	4 (10.8)
Goniometry	4 (3.5)	3 (3.8)	1 (2.7)
Muscle tone evaluation	3 (2.6)	3 (3.8)	0 (0.0)
Alberta Infant Motor Scale	3 (2.6)	1 (1.3)	2 (5.4)
Sensory Profile	3 (2.6)	1 (1.3)	2 (5.4)
Functional Independence Measure	1 (0.9)	0 (0.0)	1 (2.7)
Do you use the ICF to guide the assessment and intervention/which domains?			
No	77 (66.4)	53 (67.1)	24 (64.9)
Yes, but do not know how to describe which domains used	24 (20.7)	15 (19.0)	9 (24.3)
Yes, body functions and activity/participation	6 (5.2)	3 (3.8)	3 (8.1)
Yes, all of them	6 (5.2)	6 (7.6)	0 (0.0)
Yes, activity and participation	3 (2.6)	2 (2.5)	1 (2.7)
Which intervention techniques do you use?			
Neurodevelopmental treatment	37 (31.9)	27 (34.2)	10 (27.0)
Sensorimotor stimulation	26 (22.4)	10 (12.7)	16 (43.2)
Did not describe	23 (19.9)	16 (20.3)	7 (18.9)
Suit therapies	12 (10.3)	12 (15.2)	0 (0.0)
Sensory integration therapy	8 (6.9)	3 (3.8)	5 (13.5)
Only orientations	7 (6.0)	7 (8.9)	0 (0.0)
Kinesiotherapy	5 (4.3)	5 (6.3)	0 (0.0)
Aquatic physical/occupational therapy	5 (4.3)	3 (3.8)	2 (5.4)
Assistive technology prescription	5 (4.3)	0 (0.0)	5 (13.5)
Motor training	3 (2.6)	3 (3.8)	0 (0.0)
Proprioceptive neuromuscular facilitation	2 (1.7)	2 (2.5)	0 (0.0)
Stretching	2 (1.7)	2 (2.5)	0 (0.0)
Balance training	2 (1.7)	2 (2.5)	0 (0.0)
Respiratory physical therapy	2 (1.7)	2 (2.5)	0 (0.0)
Electrotherapy or kinesiotaping	2 (1.7)	2 (2.5)	0 (0.0)
Music therapy	1 (0.9)	0 (0.0)	1 (2.7)
Weekly frequency			
1	39 (33.6)	23 (29.1)	16 (43.2)
2	52 (44.8)	35 (44.3)	17 (45.9)
3–5	12 (10.3)	12 (15.2)	0 (0.0)
Did not inform	13 (11.2)	9 (11.4)	4 (10.8)
Intensity (hours/day)			
≤1	92 (79.3)	59 (74.6)	33 (89.2)
2	7 (6.0)	7 (8.9)	0 (0.0)
3–5	4 (3.5)	4 (5.1)	0 (0.0)
Did not inform	13 (11.2)	9 (11.4)	4 (10.8)

Abbreviation: ICF, International Classification of Functioning, Disability and Health.

## DISCUSSION

The current study investigated the practices of PTs and OTs who provided follow-up care for children with CZS in Brazil and explored whether these practices are based on scientific evidence and on the ICF model. The majority of participants were PTs, were from the Northeast of the country (the region with the highest number of CZS cases in Brazil), and worked in public rehabilitation centers. The results demonstrated that most participants did not use interventions that are based on current scientific evidence, did not use the ICF model, and did not evaluate children with CZS using standardized instruments.

Seeking to implement the use of the ICF model, in 2016, the Brazilian Ministry of Health offered training for rehabilitation professionals who work with children with CZS; however, the current study demonstrated that this implementation remains a challenge in Brazil.^19^^,^^21^ The ICF is the reference model to guide therapists in the assessment and planning of interventions and provides many complementary aspects on how we can view health and functioning when providing services.^21^^,^^24^ In contrast to a historical perspective on disability, the ICF provides a more complex picture of the child and has been used to identify appropriate goals and develop treatment plans that are meaningful to the child and family.^20^^,^^24^ Rather than being an intervention focused on functioning, which also incorporates aspects of activity, participation, and environment, the nonimplementation of the ICF model contributes to an intervention focused on disability.^25^^,^^26^

The current study showed that a small proportion of participants used standardized assessment tools, and the majority did not assess children with CZS before starting the intervention. In the area of rehabilitation, the assessment of health status (preferably all functioning domains) needs to precede any type of intervention.^24^^,^^27^ The use of standardized and validated assessment tools, together with a family-centered approach, is fundamental to identifying disabilities, facilitators, and barriers to participation; establishing relevant goals; choosing appropriate interventions; and reassessing the achievement of goals and, consequently, the intervention effect.^2^^,^^24^^,^^25^^,^^27^

The current study showed that a small proportion of participants used standardized assessment tools, and the majority did not assess children with CZS before starting the intervention.

Based on the assessments, task- and context-specific goals should be set at the appropriate level of challenge and updated regularly.^15^ Nevertheless, evidence-based assessment tools for children with disabilities are not widely used by health care professionals in daily practice.^28^^,^^29^ The literature highlights the lack of professional motivation, time, training, and support from institutions as reasons for these difficulties.^28^^,^^29^ Engagement in assessment requires a conceptual shift by therapists and organizations to understand the assessment as part of, not an adjunct to, therapy.^28^ In addition, among the assessment tools mentioned by participants, the majority focus on capacity or what a child can do in a standardized environment (e.g., Gross Motor Function Measure), performance or what a child actually does in their daily environment (e.g., Pediatric Evaluation of Disability Inventory), or body functions and structures (e.g., goniometry). None of the cited instruments assess participation. This reflects that planning of the interventions probably did not involve strategies to increase the frequency and involvement of children in recreation and leisure activities, which would be more relevant to them.^2^^,^^20^^,^^24^^,^^26^^,^^30^^,^^31^

The results show that a small portion of the participants used scientific evidence-based practice as a source of information to choose interventions, even though the majority have at least 1 specialization in the field. These findings were reflected by the choice of interventions most frequently implemented by participants that did not present evidence of a positive effect for children with CP.^14^ The literature shows that the main barriers to implementing evidence-based practice by PTs and OTs are lack of time, publication language, lack of statistical knowledge, and limited access and employer support, as well as challenges in identifying high-quality studies, notably in low- and middle-income countries.^32^^,^^33^ Evidence-based practice becomes even more challenging in newly identified health conditions and requires efforts from the scientific community.^34^ Strategies to overcome these barriers involve knowledge translation, which includes adequate financial and physical resources, management support, training, and continuing education programs.^35^

Scientific evidence-based interventions recommended for motor skills outcomes involve child-initiated movement, targeted motor training activities, task- and context-specific practice, environmental enrichment, family engagement, repetition, and intensity.^14^^,^^15^^,^^36^^,^^37^ In addition, postural management, prescription of adequate assistive technology, and hip surveillance should be implemented due to the high risk of musculoskeletal impairments presented in children compatible with Gross Motor Function Classification System levels IV and V.^14^^,^^38^ In contrast with these recommendations, the current study showed that the most prevalent rehabilitation interventions used in the Brazilian children with CZS are NDT, sensorimotor stimulation, suit therapies, and sensory integration. Therapy approaches that rely on specific therapist handling techniques and on remediating body structural and functional impairments, such as NDT and sensory integration, are not supported by current scientific evidence, and the literature recommends that they should not be implemented.^14^^,^^15^^,^^36^^,^^37^

In recent decades, the focus of PT and OT interventions for children with CP has changed from fixing impairments to promoting participation and independence.^25^^,^^26^^,^^39^ Motor learning principles support the idea that the child needs to react to sensory feedback of their own movements to produce spontaneous adaptive solutions.^37^^,^^39^ Thus, contemporary interventions are moving toward a more ecological task-oriented approach that considers the influence of the child's abilities, the demands of the task, and the context of the environment.^25^^,^^26^^,^^39^

Contemporary PT and OT interventions are moving toward a more ecological task-oriented approach that considers the influence of the child's abilities, the demands of the task, and the context of the environment.

The participants of this study frequently mentioned “sensorimotor stimulation” as an intervention; however, it was not possible to understand the components of this type of intervention or whether it incorporated principles of “top-down” (e.g., goal-directed training) or “bottom-up” (e.g., NDT) interventions.^36^ The absence of a common language and a specific definition of the rehabilitation techniques often makes it difficult to understand what is being carried out and warrants the development of clear guidelines.^13^^,^^36^ Another intervention modality that has gained notoriety in Brazil is suit therapy (i.e., PediaSuit and TheraSuit).^13^ This intervention is restricted to a small number of families due to its high cost and does not show evidence of a positive effect on participation outcomes, especially in children with more significant disabilities (e.g., CZS).^14^^,^^40^ Furthermore, due to its high cost, this type of intervention is not suitable to be offered to the population of low- and middle-income countries, such as Brazil.

Corroborating the findings of the present study, a recent scoping review regarding interventions for children with CP delivered by Brazilian PTs found that the main focus of the interventions was on reducing impairments and activity limitations, with minimal attention to participation and environmental factors.^13^ The review also identified that the most commonly investigated interventions in Brazilian studies are suit therapy and NDT.^13^ Indeed, 2 of the studies conducted in Brazil that included children with CZS investigated the effects of suit therapy^17^ and NDT,^18^ but they were not controlled studies, and their results cannot provide support for the use of these interventions.

Concerning OT approaches, recent evidence also supports family-centered practice and task-oriented approaches, such as GAME,^36^ which is based on the principles of active motor learning, family-centered care, parent coaching, and environmental enrichment.^16^^,^^41^ The GAME approach actively involves the parents and focuses on the development of the children's skills in the context in which they live, making it possible to be implemented for low-income populations.^41^ A Brazilian study using GAME in infants with CZS identified that the mothers in the GAME-based group reported significant improvements in their infants' performance and an enriched home environment.^16^ Although that study was conducted in Brazil, it does not reflect the OTs' clinical practice reported in the current study, reinforcing the gap between evidence and clinical practice and the need for knowledge translation strategies.^35^^,^^42^

The findings of this study indicate an urgent need for evidence-based assessment and intervention practices for the management of children with CZS and other causes of CP. We expect that these findings will contribute to guiding training actions for PTs and OTs that favor the implementation of evidence-based practices, guaranteeing access to better services for children with CZS and, consequently, reducing health care costs for expensive interventions that are not based on evidence. Further studies are needed to identify whether all children with CZS have access to necessary health services and the related costs of treatment.

### Limitations

This study has some limitations to be considered. The response rate could not be estimated because the total number of PTs and OTs working with CZS in Brazil was not available. Furthermore, it was not possible to estimate the necessary sample size because Brazil is a country with a vast territorial extension. Another limitation was the high rate of participants who initially agreed to take part in the study but did not answer the questionnaire. We do not have information on whether these participants actually worked with children with CZS or whether they did not participate due to the characteristics of the questions asked. The questionnaire did not include specific questions regarding barriers and facilitators for implementing evidence-based practice, hindering an in-depth analysis of these aspects. Despite these limitations, efforts were undertaken to reach as many participants as possible. This study reflects the overall challenges in professional training and service provision, which may not be exclusively related to this new health condition.

## CONCLUSIONS

This study demonstrated that the PT and OT services provided to Brazilian children with CZS are not aligned with the ICF model and evidence-based practices. Professional training, knowledge translation strategies, and adequacy for the Brazilian population context are needed to implement evidence-based practices and improve the quality of rehabilitation programs available for Brazilian children with CZS and other causes of CP.

## Supplementary Material

23-00219-Ayupe-Supplement-clean.pdf

## Notes

**Peer Reviewed**

**First published online:** December 15, 2023.
