# Supplementary material for: Rehabilitation Practices Delivered by Physical and Occupational Therapists to Brazilian Children With Congenital Zika Syndrome: A Cross-Sectional Study
Source: Glob Health Sci Pract. 2023 Dec 22;11(6):e2300219. doi: 10.9745/GHSP-D-23-00219 (PMC10749651; doi:10.9745/GHSP-D-23-00219)
Supplement: 23-00219-Ayupe-Supplement-clean.pdf [file 23-00219-Ayupe-Supplement-clean.pdf]

Supplement to: Ayupe KMA, Dias YMBC, Cazeiro APM, de Campos AC, Longo E. Rehabilitation practices delivered by physical and occupational therapists to Brazilian children with congenital Zika syndrome: a cross-sectional study. *Glob Health Sci Pract*. 2023;11(6):e2300219. https://doi.org/10.9745/GHSP-D-23-00219

QUESTIONNAIRE

Dear participant,
You are being invited to answer a Questionnaire regarding your clinical practice caring for pediatric patients, especially children with Congenital Zika Syndrome (CZS). We guarantee the preservation of your identity and the answers to this questionnaire will be the responsibility of the researchers involved, and will be used solely and exclusively for academic-scientific dissemination purposes.

Please, answer the questions below.

Personal and professional characteristics

1. Gender
2. Age ____
3. In which region of Brazil do you work?

   North □

   Northeast □

   Midwest □

   South □

   Southwest □
4. Profession

   Physical therapist □ Occupational Therapist □
5. Specify your education level:

   Bachelor's degree □

   Specialist □

   Residence □

   Master's degree □

   Master's student □

   Doctorate □

   Post-Doctorate □
6. How many years of clinical experience do you have treating children or young people with deficiency? ____

Work environment

7. Which workspace best describes where you provide services to children with CZS?

   Public Rehabilitation Centers/clinics □

   University Centers □

   Community Organization Centers □

   Private Clinics □

8. What is the funding source of your work institution/workspace?

   Public □

   Private □

   Non-governmental organizations □

   Other ____

9. Is your workplace an educational institution (defined as an institution that receives healthcare students for their clinical/training routines)?

   Yes □   No □

10. Is rehabilitation research carried out at your institution/workplace?

    Yes □   No □

11. Do you work in a service that involves a multidisciplinary team?

    Yes □   No □

12. What are the health professionals who also treat the children with CZS in your work team?

    Physicians □

    Speech therapist □

    Music therapist □

    Other ____

13. How many children with SZV are currently being followed in your workplace?

    Estimated total number of children: __________

    Estimated number of children treated weekly:____________

Rehabilitation intervention characteristics

14. Have you or another professional on your work team participated in the development of a specific rehabilitation program for children with CZS?

    Yes □   No □

15. If the answer above is yes, what were the sources of information used for the development of this rehabilitation program?

____

____

____

16. What are the sources of information that YOU use to plan the interventions that you carry out with children with CZS?

____

____

____

17. Do you use assessment tools/instruments before starting the intervention?

    Yes □ No □

18. Which ones?

____

____

____

19. Do you use periodic evaluation tools/instruments or at the end of the intervention to monitor the effect of the rehabilitation program carried out?

    Yes □ No □

20. Which ones?

____

____

____

21. Do you use the International Classification of Functioning, Disability and Health (ICF) to guide the assessment and interventions performed with children with CZS?

    Yes □ No □

22. If the answer above is yes, which ICF domains do you consider in your work processes?

____

____

____

23. What intervention techniques do you use in the treatment of children with CZS in your workspace/ institution?

____

____

____

____

____

24. In your workplace, what is the weekly frequency of consultations with children with CZS? In other words, how many times a week does the child receive care from you?

____

25. How long, in hours or minutes, does a treatment session last?

____

Thank you very much for your precious contribution!

Research team
